# Empathy and Its Relationship With Social Functioning in Individuals at Ultra-High Risk for Psychosis

**DOI:** 10.3389/fpsyt.2021.730092

**Published:** 2021-11-11

**Authors:** Daan Jan Kuis, Tara van de Giessen, Steven de Jong, Bouwina Esther Sportel, Nynke Boonstra, Rozanne van Donkersgoed, Paul H. Lysaker, Ilanit Hasson-Ohayon, Gerdina Hendrika Maria Pijnenborg

**Affiliations:** ^1^Geestelijke Gezondheidszorg (GGZ) Drenthe Mental Health Institute, Department of Psychotic Disorders, GGZ Drenthe, Assen, Netherlands; ^2^Lentis Research, Lentis Psychiatric Institute, Groningen, Netherlands; ^3^Research Group Care and Innovation in Psychiatry, NHL Stenden University for Applied Sciences, Leeuwarden, Netherlands; ^4^KieN Early Intervention Service, Leeuwarden, Netherlands; ^5^Department of Clinical and Developmental Neuropsychology, Faculty of Behavioural and Social Sciences (BSS), University of Groningen, Groningen, Netherlands; ^6^Richard L. Roudebush VA Medical Center, Indianapolis, IN, United States; ^7^Department of Psychology, Bar-Ilan University, Tel Aviv, Israel

**Keywords:** cognitive empathy, affective empathy, ultra-high risk for psychosis, schizophrenia spectrum disorder, social functioning, psychosis, Faux pas

## Abstract

**Introduction:** Social functioning is often impaired in the ultra-high-risk (UHR) phase of psychosis. There is some evidence that empathy is also impaired in this phase and that these impairments may underlie difficulties in social functioning. The main aim of this study was to investigate whether cognitive and affective empathy are lower in people in the UHR phase of psychosis in comparison to healthy controls, and whether possible impairments have the same magnitude as in people with schizophrenia. A second aim was to examine whether there is a relationship between empathy and social functioning in individuals in the UHR phase.

**Method:** Forty-three individuals at UHR for psychosis, 92 people with a schizophrenia spectrum disorder, and 49 persons without a psychiatric disorder completed the Interpersonal Reactivity Index (IRI), Questionnaire of Cognitive and Affective Empathy (QCAE), and Faux Pas as instruments to measure empathy. The Time Use survey was used to measure social functioning. MAN(C)OVA was used to analyse differences between groups on empathy and social functioning, and correlations were calculated between empathy measures and social functioning for each group.

**Results:** The UHR group presented significantly lower levels of self-reported cognitive empathy than the healthy controls, but not compared to patients with SSD, while performance-based cognitive empathy was unimpaired in the UHR group. On the affective measures, we found that people with UHR and patients with SSD had significantly higher levels of self-reported distress in interpersonal settings compared to healthy controls. In the UHR group, perspective-taking was negatively associated with time spent on structured social activities. In the SSD group, we found that structured social activities were positively associated with perspective-taking and negatively associated with personal distress in interactions with others. Lastly, in people without mental illness, social activities were positively associated with performance-based perspective-taking.

**Conclusion:** Impairments in subjective cognitive empathy appear to be present in the UHR phase, suggesting that difficulties in interpreting the thoughts and feelings of others precede the onset of psychotic disorders. This can inform future interventions in the UHR phase.

## Introduction

Subclinical psychotic symptoms can precede the onset of psychotic disorders (1, 2). These subclinical symptoms are included in the ultra-high risk (UHR) criteria, which define the characteristics of individuals who are at risk for a psychotic spectrum disorder. The criteria for establishing a UHR state consist of a decline in functioning combined with one or more of the following features: attenuated psychotic symptoms, a family history of psychotic spectrum disorder, or brief limited intermittent psychotic symptoms (3, 4). A long-term goal of identifying people with UHR is to delay or prevent the onset of psychosis (5).

Despite significant research efforts, however, the transition rate to psychosis in the UHR group based on current criteria is low to moderate (5), with recent studies showing a transition rate of 20% over 2 years (6–9). Statistical models combining current criteria with information on negative symptoms and social functioning do not perform much better: one recent individual participant data meta-analysis (IPD-MA) of data from 1,676 individuals at high clinical risk found the model reached only moderate prognostic performance (10). The current criteria are therefore lacking in specificity, and a more accurate prediction of transition is needed to reduce the high number of false positives.

It is possible that adding information from different markers of functioning could improve predictive models (10). A promising marker is impaired empathy, which is often observed among this population (11). Decety and Jackson (12) defined empathy as “the ability to appreciate the emotions and feelings of others with a minimal distinction between self and other.” Empathy is often divided into two components: cognitive and affective empathy (13). Cognitive empathy is the ability to interpret the thoughts and feelings of other people using contextual information (14, 15). In contrast, affective empathy is often referred to as the ability to share the emotional experience of another person (12, 15), and as such, it enables people to feel vicariously what others feel (15) and leads to compassion about others' emotional state (16). Both the cognitive and affective aspects of empathy make it possible to take the perspective of another person and to understand another person's feelings, thoughts, and motivations (17).

Both in first-episode patients and in patients with chronic schizophrenia, research shows that cognitive empathy is impaired compared to people in the general population (18–20). Impaired empathy is also visible in remitted patients, suggesting that deficits in cognitive empathy could be a characteristic of the disorder (20). In addition, impaired cognitive empathy is independent of the progression of the illness after the first episode of psychosis, meaning that cognitive empathy does not seem to decline further after the onset of the first psychotic episode (18). In the UHR phase, there is some evidence that cognitive empathy is already less than in the general population (11, 18). When cognitive empathy was measured with performance-based instruments, persons in the UHR group showed significant impairment in cognitive empathy, although to a lesser extent than the impairment found in first-episode patients and patients with schizophrenia (11, 18, 21). However, more research is needed because of inconsistent results for self-report measures, the use of different measurement instruments, and small sample size (11, 18).

As mentioned above, affective empathy, mainly measured by self-report instruments, is also impaired in psychosis spectrum disorders (22–24). One recent large-scale study found impaired performance-based affective empathy in people with UHR (25). Besides this study, research on affective empathy in UHR is lacking, to the best of our knowledge.

A possible important consequence of impaired empathy is the associated decrease in social functioning (26, 27), which is considered a key feature of psychosis spectrum disorder. Studies have shown that social cognitive processes, which include the cognitive elements of empathy, are critical for social functioning, even more so than the presence of positive symptoms (28–30). Both in patients with chronic schizophrenia and in first-episode patients, impairment in cognitive empathy is associated with problems in social functioning (26, 27, 31), although this association is not always found, and when associations are found (32, 33) a lot of variance remains unexplained (31). To the best of our knowledge, studies on the relationship between affective empathy and social functioning in schizophrenia spectrum disorders are lacking.

Social functioning declines before the onset of the first episode, during the UHR phase, and then decreases further around the first psychotic episode (30, 34, 35). It is still unclear which factors contribute to impaired social functioning in the UHR phase (36). There is some evidence suggesting that impaired social cognition, which includes cognitive empathy, may underlie impairments of social functioning in the UHR phase (36).

The main aim of this study was to investigate whether cognitive and affective empathy are affected in people in the UHR phase when compared to a sample from the general population without a psychiatric disorder and a more chronic group of patients with a diagnosis of schizophrenia. For this aim, a UHR sample was compared to a sample of people from the general population and to a group of people with a schizophrenia spectrum disorder (SSD).

The second aim was to explore the relationship between empathy and social functioning in individuals in the UHR phase, persons with a SSD diagnosis, and people without mental illness. Regarding the first research question, we hypothesize that both cognitive and affective empathy are affected in UHR, although to a lesser extent than impairments in people with schizophrenia spectrum disorder. With regard to the second question, our hypothesis is that there is a positive relationship between empathy and social functioning in the UHR phase and in SSD. We do not have a specific hypothesis about this relationship in healthy controls.

## Methods

### Sample

Forty-three help-seeking patients with UHR status [validated using the CAARMS interview (37)], aged between 15 and 35 years old and receiving mental health care participated in this study. We also included 92 patients diagnosed with a schizophrenia spectrum (SSD) disorder, as well as a general population control sample of 49 people (for demographics, see Table 1). The people diagnosed with SSD and most of the general population controls were recruited as part of a randomized clinical trial from another study, the MERIT study (38, 39). None of the general population controls had a history of psychiatric disorders.

**Table 1:** Demographic variables ultra-high risk, schizophrenia spectrum disorder, and general population controls.

**Variable**	**UHR group** ***n* = 43**	**SSD group** ***n* = 92**	**GPC** ***n* = 49**
Gender (% male)	44	66	71
	**Mean (sd)**	**Mean (sd)**	**Mean (sd)**
Age	22.1 (5.8)^*^	38.9 (11.0)	36.1 (14.0)

* *UHR different from SDD and GPC, p < 0.05*.

Given that patients with SSD included in the MERIT sample were people with chronic schizophrenia and thus somewhat older, the controls were relatively old (*M* = 39.4, *SD* = 13.0). Therefore, eight additional younger healthy controls were recruited separately to allow for comparison with the much younger UHR group.

People with a UHR were recruited from two mental health care services, GGZ Drenthe and GGZ Friesland, in the Netherlands. All newly referred patients (except those who already had an SSD) were invited to fill out the Prodromal Questionnaire 16 as a part of routine assessment at the start of treatment [PQ-16; (40)]. Patients with a score of 6 or higher were invited for further assessment. The Comprehensive Assessment of At Risk Mental State [CAARMS; (37)] was used to determine whether the UHR criteria were met. Exclusion criteria were co-morbid neurological pathology, severe drug abuse/substance dependence, or an estimated IQ score <70.

Participants in the SSD group were recruited from six mental health care institutions in the Netherlands (GGZ Drenthe, GGZ Friesland, University Medical Center Groningen, Lentis, Yulius, and Dimence), as part of the MERIT study (38). Exclusion criteria were: current psychotic episode (PANSS, positive symptoms average >4), IQ <70, age <18, not being able to give informed consent, medication change in the 30 days prior to assessment and comorbid neurological disorder. Diagnosis was confirmed using the Mini International Neuropsychiatric Interview (41).

The general population group reported they had never received a psychiatric diagnosis nor received treatment for mental health problems. They were recruited through social media channels, local schools and flyers in the area of the mental health care centers.

### Measures

#### Empathy Measures

**Interpersonal Reactivity Index** [IRI; (42)]: The IRI is a self-report questionnaire with 28 items divided into four subscales measuring two dimensions of empathy (current study Cronbach's α = 0.82). Cognitive empathy is measured by the sub-scales perspective taking (α = 0.72) and fantasy (α = 0.75). The subscales empathic concern (α = 0.68) and personal distress (α = 0.78) measure affective empathy. Each subscale contains seven items. Participants have to determine the extent to which each statement describes them and rate each item on a five-point Likert scale (from 0—*does not describe me well* to 4—*describes me very well*). Higher scores indicate higher empathy, all four IRI subscales were used separately in the analysis.

**Questionnaire of Cognitive and Affective Empathy** [QCAE; (15)]: The QCAE (current study Cronbach's α = 0.83) consists of 31 items and is designed to measure self-reported cognitive and affective empathy. The questionnaire is divided into five subscales: the cognitive empathy scale (α = 0.85) comprises two subscales, Perspective Taking and Online Simulation. The other three subscales, Emotion Contagion, Proximal Responsivity and Peripheral Responsivity, assess affective empathy (α = 0.72). Participants used a five-point Likert scale (from 4—*strongly agree* to 0—*strongly disagree*), with higher scores indicating higher empathy. The QCAE has good validity and internal consistency (15).

**Faux Pas Task** (43, 44): This test is used to assess performance-based cognitive empathy. Ten stories were read aloud by the experimenter, and participants were asked whether anyone in the story said something awkward (Faux Pas cognitive) and whether the remark made other people in the story feel sad and embarrassed (Faux Pas affective). Both subscales measure cognitive empathy. Five stories contain a faux pas and five control stories do not. One point was awarded for each test question answered correctly. All scores were added up to give a total score, with a higher scores indicating higher cognitive empathy.

#### Social Functioning

**Time Use Survey** [TUS; (45)]: This semi-structured interview investigates how the participant has spent his or her time over the last month. A shortened version of the interview was used (45), which took ~20 min to complete (inter-rater reliability ICC = 0.99). The TUS gives a direct measure of time spent in structured activities, such as employment, education and training, voluntary work, leisure and sport activities, hobbies, socializing, resting, sleep, child care, and housework and chores.

Respondents were asked how many times they had been busy with each activity over the past month and for how long on each occasion. A weekly average in minutes was then calculated for each activity category. A composite score of hours per week spent in constructive economic activity (paid/voluntary work, education, household chores, and childcare) and structured activity (constructive economic activity plus leisure activities, sports, and hobbies) were calculated. The TUS is considered a good proxy for measuring social functioning, since it not only measures time spent on constructive economic activity but also other forms of activity, capturing the whole spectrum of activities that are considered part of social functioning (34). The TUS has been used in previous research with people with schizophrenia and was found to be feasible and acceptable (34).

### Procedure

When the patients met the UHR criteria, they were informed about the current study and asked to participate. After a complete description of the study, all participants (and parents of participants <18 years) gave written informed consent and granted permission to use their data for further research.

Approval for the assessment of the patients (SSD and UHR) was given by the local medical Ethics Committee (numbers METc2013.124 and METc2014.279) and for the comparison group by the ethical committee of the Psychology Department at the University of Groningen (ECP research code: ppo-013-109). Assessments were conducted by trained assessors with at least a BSc in psychology.

### Data Analyses

Statistical analyses were performed using SPSS 23.0 and the level of significance was set at *p* < 0.05. Analysis assumptions were checked for total scores as well as subscales. We removed one outlier in the group with schizophrenia with a large discrepancy on empathy measures. Removing this outlier did not change the results. No violations of assumptions were found.

First, baseline demographic characteristics were generated and compared. Second, to test whether empathy was significantly different in the UHR group compared to the schizophrenia group and healthy controls, a multiple analysis of (co)variance [MAN(C)OVA] was performed to assess associations between scores on empathy scales and social functioning. Due to the significant between-group differences, we adjusted for age and gender. We performed a MAN(C)OVA on the IRI and QCAE subscales and one on the Faux Pas subscales. To avoid having to exclude participants due to missing data, analyses were conducted separately.

Subsequent analyses (ANOVA) were used to compare between-group differences on empathy and social functioning scores, with *post-hoc* comparisons using Tukey *post-hoc* tests that control for Type I error rate. The exception was the IRI subscale of empathic concern. For this scale, we used Tamhane's T2 test because the homogeneity assumption on this scale was not met. Effect sizes are reported as Cohen's *d* (46).

Third, group differences in the Time Use Survey were evaluated with a one-way analysis of variance. Fourth, within the UHR group, we evaluated correlations (Pearson correlation) between empathy measures and social functioning.

## Results

### Participant Characteristics

Descriptive statistics of the three groups are shown in Table 1. Differences were found in age and gender between the UHR group and the control group and between the UHR group and the schizophrenia group. On demographic variables, no differences were found between the SSD group and the control group.

### Group Differences in Empathy Measures

**Figure 1:**
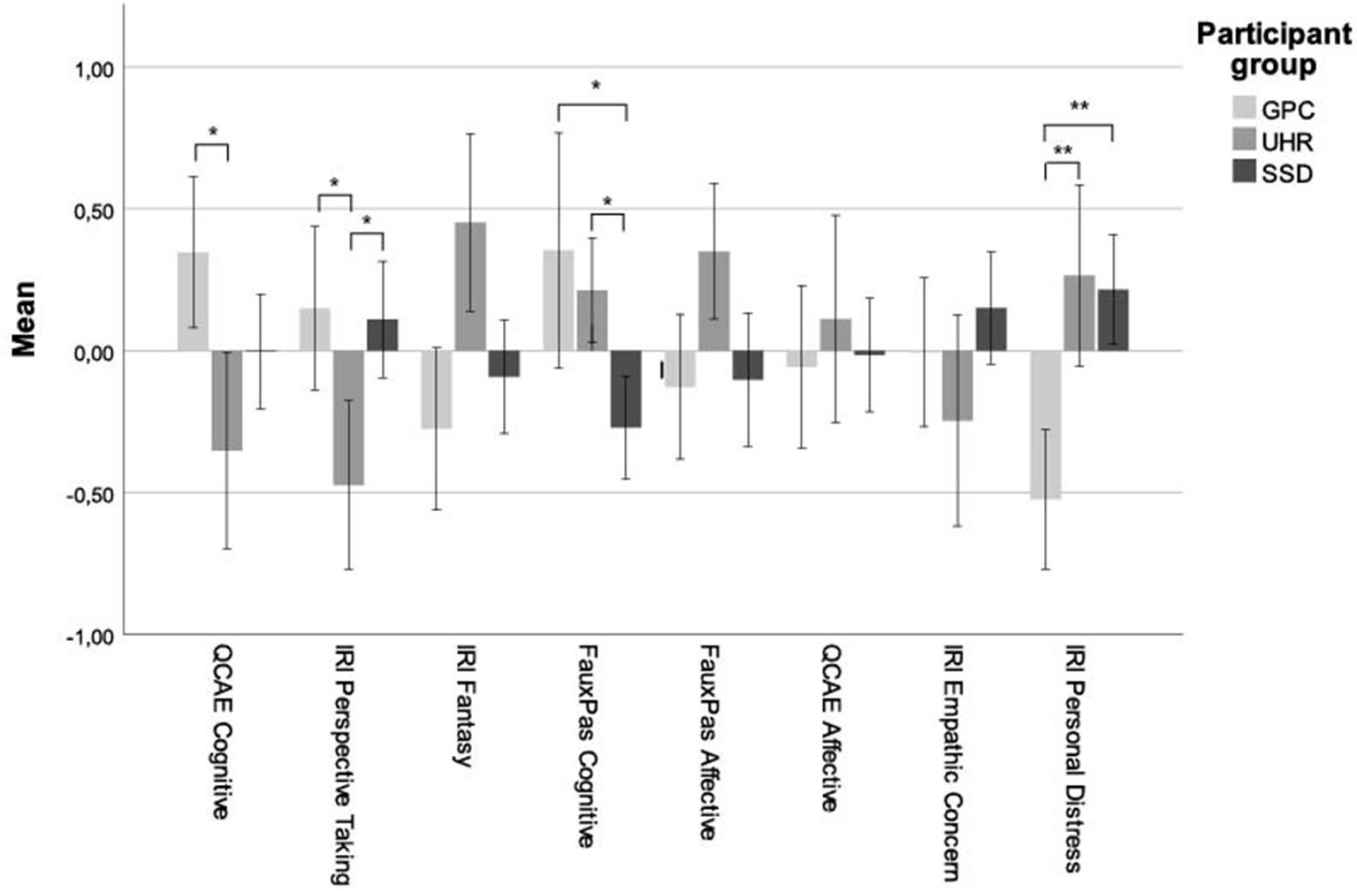
Group differences on all empathy measures displayed in *z*-scores. ^*^= 0.05, ^**^= 0.001.

Two MANOVAs were conducted to determine group differences in all three empathy measures. There was a statistically significant difference between the groups in the dependent variables. Wilks' Lambda test showed a significant effect of group on the empathy measures QCAE and IRI [∧ 0.696, *F*_(12, 352)_ = 5,826, *p* < 0.001] and Faux Pas [∧ 0.880, *F*_(4, 348)_ = 5.362, *p* < 0.001], meaning groups differed on one or more of the empathy measures. The results are displayed in *z*-scores in Figure 1.

#### Cognitive Empathy

Univariate testing using ANOVA showed significant group differences on the IRI subscales perspective-taking and personal distress and on the QCAE cognitive subscale and the Faux Pas cognitive subscale (see Table 2).

**Table 2:** Univariate group comparisons on empathy between ultra-high risk, schizophrenia spectrum disorder, and general population controls with covariates age and gender.

**Variable**	**UHR group** ***n* = 43**	**SSD group** ***n* = 92**	**GPC** ***n* = 49**	**Statistical parameters, *F*_**(df)**_, *p***
	**Mean (sd)**	**Mean (sd)**	**Mean (sd)**	
IRI total (range 0–112)	60.86 (14.93)	61.95 (13.59)	56.41 (12.78)	*F*_(2, 179)_ = 2.758, *p* = 0.066
IRI perspective taking (C)^a^^,^^b^ (range 0–28)	13.70 (4.74)	16.54 (4.80)	17.08 (4.66)	*F*_(2, 179)_ = 5.687, *p* = 0.004
IRI fantasy (C) (range 0–28)	16.98 (5.92)	13.77 (5.55)	13.14 (5.59)	*F*_(2, 179)_ = 2.547, *p* = 0.081
IRI empathic concern (A) (range 0–28)	16.14 (5.57)	17.96 (4.34)	17.00 (4.01)	*F*_(2, 179)_ = 1.434, *p* = 0.241
IRI personal distress (A)^a^^,^^c^ (range 0–28)	14.05 (5.81)	13.67 (5.27)	9.18, (4.60)	*F*_(2, 179)_ = 12.582, *p* > 0.001
QCAE total (range 31–124)	86.35 (11.28)	88.32 (10.27)	90.82 (9.20)	*F*_(2, 179)_ = 1.596, *p* = 0.206
QCAE cognitive empathy (C)^a^ (range 19–52)	52.51 (8.81)	55.17 (7.58)	57.86 (6.55)	*F*_(2, 179)_ = 3.553, *p* = 0.031
QCAE affective empathy (A) (range 12–48)	33.84 (6.21)	33.14 (4.99)	32.96 (4.77)	*F*_(2, 179)_ = 0.077, *p* = 0.926
Faux pas cognitive (C)^b^^,^^c^ (range 0–10)	9.16 (0.95)	8.40 (1.38)	9.39 (2.16)	*F*_(2, 173)_ = 7.328, *p* = 0.001
Faux pas affective (A)^a^^,^^b^ (range 0–10)	2.93 (1.14)	2.26 (1.66)	2.23 (1.24)	*F*_(2, 173)_ = 1.998, *p* = 0.139

a *UHR different from general population controls, p < 0.05*.

b *UHR different from SSD, p < 0.05*.

c *SSD different from general population, p < 0.05*.

*Post-hoc* testing using Tukey's test revealed significant differences between the UHR group and general population controls and the SSD group; the IRI subscale Perspective Taking showed significantly lower scores for the UHR group compared to general population controls (mean difference = −3.38, *p* = 0.002, *d* = 0.72) and SSD (mean difference = −2.85, *p* = 0.004, *d* = 0.59).

On the QCAE cognitive subscale, the UHR group scored significantly lower compared to the general population controls (mean difference = −5.35, *p* = 0.003, *d* = 0.69). There was no significant difference between the UHR group and the SSD group.

On the Faux Pas cognitive scale, the UHR group (mean difference = 0.77, *p* = 0.020, *d* = 0.64) and the general population controls (mean difference = 0.99, *p* = 0.002, *d* = 0.55) showed significantly higher scores compared to SSD. No differences were found on the Faux Pas Affective subscale.

As a whole, these analyses on cognitive empathy showed that self-reported perspective-taking was the worst in the UHR group, with a mean score significantly lower than both other groups. The UHR group scored comparably to the SSD group (and lower than controls) on self-reported empathy as a whole, however performance-based cognitive empathy in the UHR group was comparable to the controls and better than in the SSD group.

#### Affective Empathy

As shown in Table 2, univariate testing using ANOVA showed significant effects of group on the affective empathy measures of IRI Personal Distress.

*Post-hoc* testing revealed that on the IRI Personal Distress scale, SSD patients (mean difference = 4.49, *p* < 0.001, *d* = 0.91) and the UHR group (mean difference = 4.86, *p* < 0.001, *d* = 0.93) showed significantly higher levels of distress compared to healthy controls. No significant results of the group were found on the IRI Empathic Concern and QCAE affective subscales.

In summary, affective empathy was not impaired in the UHR and SSD groups, with the exception of UHR and SSD groups reporting more personal distress than the control group.

### Relationship Between Empathy and Social Functioning

#### Social Functioning Comparison Between Groups

To establish whether social functioning was impaired in the UHR group, we examined how much time the participants had spent on different activities over the last month and made a comparison between the three groups. The TUS is divided into constructive economic activities (paid/voluntary work, education, household chores, and childcare) and structured activities (constructive economic activities plus leisure activities, sports, and hobbies).

As shown in Table 3, *post-hoc* comparisons showed that the two clinical groups had significantly lower levels of time spent on constructive economic activity (UHR: mean difference = −12.1, *p* = 0.001, *d* = 0.65, SSD: mean difference = 29.1, *p* < 0.001, *d* = 1.30) compared to the general population control group. The SSD group also showed significantly lower levels of constructive economic activity compared to the UHR group (mean difference: −11.9, *p* < 0.001, *d* = 0.52). The UHR group took a mid-position between the healthy control group and SSD patients. Adding age and gender as covariates did not change the effect of the group.

**Table 3:** Time Use Survey (TUS), time spent on activities, in hours per week, ultra-high risk, schizophrenia spectrum disorder, and general population controls.

**Variable**	**UHR group** ***n* = 43**	**SSD** ***n* = 92**	**General population controls** ***n* = 49**	**Statistical parameters** ***F*_**(df)**_, *p***
	**Mean (sd)**	**Mean (sd)**	**Mean (sd)**	
TU constructive economic activity^a^−^c^	36.4 (26.8)	24.5 (18.2)	53.5 (25.9)	*F*_(2, 169)_ = 24.42, *p* < 0.001
TU structured activity^b^^,^^c^	72.4 (40.2)	41.5 (24.3)	77.4 (38.1)	*F*_(2, 169)_ = 22.51, *p* < 0.001

a *UHR different from general population control group, p < 0.05*.

b *UHR different from SSD group, p < 0.05*.

c *SSD group different from general population control group, p < 0.05*.

For Structured Activity, there was a significant difference between the SSD and general population control groups (mean difference = −35.9, *p* < 0.001, *d* = 1.12) and between the SSD and UHR group (mean difference = −30.9, *p* < 0.001, *d* = 0.93). We found no difference between the UHR group and the general population controls. The UHR group performed significantly more structured activities than SSD patients. Adding age and gender as covariates did not influence the effect of the group.

#### Correlations Between Empathy Measures and Social Functioning

In the UHR group, both the IRI fantasy subscale and the cognitive scale of the Faux Pas test had a significant moderate negative correlation with Time Use constructive economic activity (*r* = −0.33, *p* = 0.03; *r* = −0.36, *p* = 0.02). Time Use structured activities had a significant moderate negative correlation with the perspective-taking scale of the IRI (*r* = −0.41, *p* = 0.007), the fantasy scale of the IRI (*r* = −0.30, *p* = 0.05), and the Faux Pas Cognitive Scale (*r* = −0.379, *p* = 0.01). The SSD group showed a significant moderate and negative correlation between Time Use Constructive economic activities and Time Use Structured Activities and the Personal Distress Scale of the IRI **(***r* = −0.24, *p* = 0.03; *r* = −0.27, *p* = 0.013). Moreover, the IRI Perspective Taking Scale was positively associated with Time Use Structured Activity (*r* = 0.24, *p* = 0.04), which was the opposite of the finding in the UHR group. In the general population control group, only the cognitive subscale of the Faux Pas test was positively and moderately associated with the structured activity scale of Time Use (*r* = 0.44, *p* = 0.003).

## Discussion

This study compared cognitive and affective empathy in a group of individuals at ultra-high risk for psychosis with a group of people with a schizophrenia spectrum disorder and people without mental illness. Moreover, the potential correlates of both forms of empathy with social functioning were explored in all three groups. The results confirm that individuals who are at ultra-high risk for psychosis have some impairment in empathy compared to people without a psychiatric disorder, particularly in the domain of cognitive empathy. The UHR group performed less structured social activities than the people without a psychiatric disorder but more than people with SSD. In the UHR group, perspective-taking was negatively associated with time spent on structured social activities. In the SSD group, we found that structured social activities were positively associated with perspective-taking and negatively associated with personal distress in interactions with others. Lastly, in people without mental illness, social activities were positively associated with performance-based perspective-taking.

As anticipated in light of previous research (11), the UHR and SSD groups demonstrated equivalent levels of self-reported perspective-taking and general cognitive empathy, both of which were lower than the group without mental illness. By contrast, performance-based cognitive empathy in the UHR group was at the level of people without mental illness and was significantly better than in people with SSD. These results support the idea that self-reported cognitive empathy has already deteriorated in patients in the UHR phase and that these impairments are comparable to those found in schizophrenia patients (27, 47–49). However, a discrepancy was observed between the self-reported subjective perception of empathy and actual performance on a task measuring cognitive empathy such that the UHR group reported experiencing difficulties in cognitive empathy, but these difficulties did not have an impact on actual performance. Thus, although people in the UHR phase reported subjective impairments, these impairments were not detected by neuropsychological tests. This suggests that while in the UHR phase people can, at least under structured circumstances and clear instructions, still function at the level of people without mental illness, even when they may already show impairments in less structured and/or complex situations in daily life.

For affective empathy, we did not find severe impairments in the UHR phase, with the exception of more interpersonal distress in both people in the UHR phase and people with a SSD. This suggests that while cognitive empathy is impaired in the UHR phase, affective empathy is relatively spared over different phases of psychotic disorders. This may be due to the fact that cognitive empathy requires more effort, while affective empathy does not require extensive cognitive processing and instead relies on vicariously sharing emotions with others (17). There is a large body of evidence that suggests cognitive processes that require effort are especially impaired in SSD (50). This finding, coupled with the idea that affective empathy requires less cognitive effort, may explain why both people with UHR and SSD did not report impairments in this domain. People with SSD are just like others, affected by the emotions of people in their environment, and may be more distressed by these emotions than people without mental illness.

A study by Montag et al. (51) showed that subjective perspective-taking was significantly affected by duration of illness, suggesting that the cognitive component of empathy could be less affected in the early stages of the illness and may become more impaired as the illness progresses. Our findings seem to contrast a recent study on cognitive and affective empathy in the UHR phase that found impaired self-reported affective empathy in contrast to relatively intact cognitive empathy (25). This difference could be due to the fact that Montag et al. only included a performance-based assessment of cognitive empathy, on which we did not find impairments in the UHR group either. Striking, however, is still the fact that the UHR group in our sample reported lower perspective-taking than the SSD group. The UHR group is, by definition, very heterogeneous due to the low specificity offered by the criteria. As such, using such criteria will also “pick up” persons with other mental difficulties. For instance, one study reported that 20% of their UHR sample was later diagnosed with a (co-morbid) autism spectrum disorder [e.g., (52)], while the sample of another study included a large proportion of persons with a personality disorder (25). These differences, and differences in other relevant characteristics, such as personality or social resources, may contribute to inconsistent findings with regard to empathy. An alternative explanation for the fact that our UHR group reported worse cognitive empathy than SSD and controls could be that perspective-taking develops during adolescence with maturation of the prefrontal lobe and is temporarily lower in younger individuals than in adulthood (53).

As mentioned above, the additional results of the current study showed that both clinical samples reported more interpersonal distress than people without a diagnosis of mental illness. This means that people from the UHR group and the SSD group experienced more feelings of discomfort while being in contact with other people. The subscale of personal distress of the IRI measures “self-oriented” feelings of personal anxiety and unease in tense interpersonal settings (42). Davis (42) found that persons with higher levels of personal distress were shyer, experienced more social anxiety, and were less extraverted. Higher scores in interpersonal distress have been found in people with SSD and first-episode psychosis compared to people without mental illness in previous research (19, 54). One explanation for the higher scores on interpersonal distress is that people with a psychosis spectrum disorder show difficulty with emotion regulation and managing arousal (55). It has been argued that higher levels of self-oriented personal distress reflect a defect in emotion regulation rather than impaired affective empathy (19), which would imply that people in the UHR phase may have problems with emotion regulation and managing arousal rather than feeling what others feel.

Normal affective empathy in UHR is in line with results found in people with schizophrenia and first-episode patients (19, 27), suggesting that basic empathic abilities, such as affective empathy, are less affected than affective domains that require cognitive effort (47, 51).

Our results show that while in the UHR phase, people spend more time on constructive economic activities (activities related to work and education) than people with a SSD; however, they performed less constructive economic activities than people without a mental illness, which could also be related to age differences between groups. The level of structured leisure activities in the UHR group in the current study was again higher than in the SSD group, and did not deviate from that of people without mental illness. This latter finding is in contrast with previous research showing that people in the UHR phase spend less time on both economic and leisure activities than do people without mental illness (34). As mentioned above, this illustrates that UHR samples in the literature may differ in several basic features, and that these basic differences between samples should be taken into account when interpreting research findings. Of note, on both subscales of Time Use, people without mental illness spent more time on activities compared to patients with SSD. The poor functioning in the SSD group is in line with a large amount of research showing broad functioning problems (28–30), while relatively good social functioning in this specific UHR sample contrasts with previous studies showing impaired social functioning in the UHR phase (30, 35, 36).

As anticipated, we found correlations between empathy and time spent on social activities in the UHR group. In particular, perspective-taking abilities (both performance-based and self-reported) were found to be negatively associated with structured social activities. These results seem counterintuitive, suggesting that with better perspective-taking abilities, people have less structured social activities. It may be that when people in the UHR phase report good subjective perspective-taking, they are in fact over-mentalizing in the sense that they make over-interpretations of the mental states of others. This tendency has been documented before in SSD, and was associated with delusion (56). Thus, perhaps an over-interpretive perspective-taking style in the UHR group, in combination with the heightened interpersonal distress we found, may make people uncomfortable in the presence of others. This may result in more avoidance of social situations in people who report higher subjective perspective-taking. Moreover, fantasy was negatively associated with both economic and leisure activities. The less patients reported the tendency to transpose themselves into fictional characters, the more time they spent on structured social activities. It could be that people with more vivid imaginations have less need for social contact and external stimuli. The negative association between self-reported perspective-taking and social activities found in the current study in the SSD group is in line with previous studies reporting that better performance-based perspective-taking was associated with more social activities (31). Moreover, the more personal distress people with SSD reported, the less social activities they performed. The fact that empathy was not associated with economic activities may be due to the high unemployment rates in this group. Lastly, in people without mental illness, social activities were positively associated with performance-based perspective-taking.

There are several limitations to this study. The three groups differed in age and gender, which may partly explain the effects we found. In this research, we used general population control data and schizophrenia patient data from an earlier study (38, 39) in which healthy controls were matched on age and gender based on the characteristics of the patient group. The UHR group, however, was recruited later and was not matched. It included a much younger participant group compared to the healthy control group. In addition, the gender in the UHR group was much more equally distributed compared to the healthy control group. It is plausible to assume that age and gender affect empathy, with women and younger ages usually showing better performance on empathy tasks (53, 57, 58). It should be noted that controlling for age and gender in the analyses did not change the outcomes of the current study.

An additional limitation is the lack of a performance-based measure of affective empathy. Previous literature has shown that people with schizophrenia perceive themselves as more empathic than their performance on tests reflects (22). Horan et al. (59) call this the belief-ability gap. Nezlek et al. (60) showed that people are more empathic when they experience stronger affect and when they are more socially active. Performance based measurements are more suitable for capturing performance-based affective empathy than self-report. A possible instrument that might be used in future research is the Empathic Accuracy Test [EAT; (61, 62)]. This is a performance-based instrument that measures affective empathy, requiring rating of affect in people talking about something they previously experienced during brief vignettes. The EAT does not require trained clinicians to administer it, and previous research has shown that it measures empathy in an ecologically valid way (63).

A methodological limitation concerns the TUS, which is a very general measure of social functioning that assesses only the amount of time spent on social activities. For future research on empathy and social functioning, we suggest using instruments that are more sensitive to capturing the quality of interactions with other people. Previous research on patients with psychosis spectrum disorder used, for example, the Social Skill Performance Assessment (64, 65) developed by Patterson et al. (66).

With these limitations in mind, the current study showed evidence that aspects of cognitive empathy are, to some extent, already impaired in the UHR phase, indicating that difficulty interpreting the thoughts and feelings of others is present in this phase, and that cognitive empathy shows a negative association with structured social activities. The discrepancy between performance-based and self-report measures may indicate that while performance is still adequate, it requires more effort. Therefore, both self-reporting and the objective assessment of empathy should be taken into account in clinical assessment. After replication results may have important implications for treatments. For patients with UHR, it is important to provide opportunities in treatment settings in which they can experience and practice taking the perspective of others and exploring and adjusting their interpretation of social situations. When personal distress and anxiety prevent them from doing this, interventions are desirable. For example, offering training in perspective-taking by either cognitive behavioral therapy or social cognition training may improve social functioning in the UHR phase.

## Data Availability Statement

The raw data supporting the conclusions of this article is available from the authors upon request.

## Ethics Statement

The studies involving human participants were reviewed and approved by Medical Ethics Committee University Medical Center Groningen, Groningen, the Netherlands. The patients/participants provided their written informed consent to participate in this study.

## Author Contributions

RD, SJ, IH-O, PL, and GP designed the study and wrote the application for medical ethical approval. DK, TG, and BS performed the statistical analysis. DK and TG wrote the first draft of the manuscript. All authors contributed to manuscript revision, read, and approved the submitted version.

## Funding

Funding was received from Fonds NutsOhra for the original RCT in the Metacognitive Reflection and Insight Therapy (MERIT) for patients with schizophrenia study (11, 39). Fonds NutsOhra had no influence on the study or dissemination of the results.

## Conflict of Interest

The authors declare that the research was conducted in the absence of any commercial or financial relationships that could be construed as a potential conflict of interest.

## Publisher's Note

All claims expressed in this article are solely those of the authors and do not necessarily represent those of their affiliated organizations, or those of the publisher, the editors and the reviewers. Any product that may be evaluated in this article, or claim that may be made by its manufacturer, is not guaranteed or endorsed by the publisher.
